# Relationship Between Electrical Instability and Pumping Performance During Ventricular Tachyarrhythmia: Computational Study

**DOI:** 10.3389/fphys.2020.00220

**Published:** 2020-03-24

**Authors:** Da Un Jeong, Ki Moo Lim

**Affiliations:** Computational Medicine Lab, Department of IT Convergence Engineering, Kumoh National Institute of Technology, Gumi, South Korea

**Keywords:** ventricular tachyarrhythmia, action potential duration, dominant frequency, phase singularity, filament, computational study, stochastic model

## Abstract

There are representative electrical parameters for understanding the mechanism of reentrant waves in studies on tachyarrhythmia, namely the action potential duration (APD), dominant frequency, phase singularity, and filament. However, there are no studies that have directly identified the correlation between these electrophysiological parameters and cardiac contractility. Therefore, we have identified individual and integrative correlations between these electrical phenomena and contractility during tachyarrhythmia by deriving regression equations and also investigated the electrophysiological parameters affecting cardiac contractility during tachyarrhythmia. We simulated ventricular tachyarrhythmia with 48 types of electrical patterns by applying four reentry generation methods and changing the electrical conductivity of the potassium channel, which has the greatest effect on ventricular tissue. The mechanical responses reflecting electrical complexity were obtained through deterministic simulations of excitation–contraction coupling. We used the stroke volume and amplitude of myocardial tension (ampTens) as the variables representing contractility. We derived stochastic models through single- and multivariable regression analyses to identify the electrical parameters affecting contractility during tachyarrhythmia. In single-variable regression analysis, the APD, dominant frequency, and filament, excluding phase singularity, have statistically significant correlations with the stroke volume and ampTens. Among them, the APD has the maximum influence on these two mechanical parameters (standard beta coefficient: 0.859 for stroke volume, 0.930 for ampTens). The stochastic model using all four electrical parameters fails to accurately predict contractility owing to the multicollinearity between the APD and dominant frequency. We have rederived the multi-variable stochastic model using three electrical parameters without the APD. The filament has the greatest effect on the stroke volume stochastically (standard beta coefficient: 0.853 and 0.752). The dominant frequency has the greatest effect on ampTens statistically (standard beta coefficient: −0.813). We conclude that among the electrical parameters, the APD has the highest individual influence on mechanical contraction, and the filament has the highest integrative influence in both statistical terms.

## Introduction

Tachyarrhythmia causes myocardial tissue to asynchronously contract at high frequencies resulting from electrical reentry of excitation waves (Kuklik et al., 2017). Unlike the regular rhythm of a normal heart, reentrant waves from complex electrical patterns that generate a fast rhythm in a specific area of the tissue (Bray et al., 2001). These complex electrical patterns play an important role in the development and maintenance of tachyarrhythmia (Clayton and Holden, 2002). Therefore, many attempts have been made to understand the occurrence and maintenance mechanisms of tachyarrhythmia via the analysis of reentrant waves.

To date, research on tachyarrhythmia has focused on the cases of reentrant wave generation and the fibrillation mechanism by analyzing the spatial properties of reentrant waves (Karma, 1994). In studies concerning tachyarrhythmia, researchers typically employ an action potential that provides information regarding the electrical activity of the heart, which is used to analyze the mechanism of the electrical patterns in myocardial tissue. The action potential duration (APD) refers to the period during which the myocardial cell is excited and returns to a stable state. The APD provides quantitative electrical information that can be confirmed when an abnormality occurs in the membrane current through the ion channel of the myocardial cell, and it directly affects the contraction performance of the heart (Hille, 1978). The ion channel that has the greatest effect on the APD of the cells is the potassium channel. A Gain of function or loss of function in the potassium channel affects the repolarization time of the action potential, leading to prolonging or shortening of the APD (Ravens and Cerbai, 2008). Under conditions with prolonged or shortened APDs, reentrant waves can be easily induced even at normal rhythms. Furthermore, because the APD of the myocardial cells around the rotor of reentry is phenomenologically short, it can provide useful information for predicting and analyzing the electrical activity of myocardial tissue (Ten Tusscher and Panfilov, 2006; Delmar and Anumonwo, 2009). Therefore, at the cellular level, the APD can approximatively predict the electrical patterns to some extent; conversely, it can phenomenologically reflect the electrical patterns at the tissue level. In both respects, the local APD in myocardial tissue may be an important indicator for predicting electrical patterns at the tissue level.

In addition, there are distributions of dominant frequencies, phase singularities, and filaments that can serve as phenomenological indices that reflect the electrical complexity of myocardial tissue. The dominant frequency is the frequency with the highest energy in all spectra of the myocardial signal. The highest energy in the membrane potential signal produced by myocardial cells belongs to the frequency corresponding to the generation rate of the action potential. Therefore, the dominant frequency in the membrane potential signal indicates the generation rate of the action potential. By analyzing the distribution of the local dominant frequencies of myocardial tissue, we can predict the extent to which the heart will asynchronously repeat excitation and relaxation cycles. Asynchronous electrical excitation can be quantified by the distribution of the changes in the excitation rate. Therefore, the degree of asynchronous electrical excitation can be quantified through the dominant frequency distribution obtained by frequency analysis of the membrane potential signals of myocardial cells. In the reentrant waves observed at the time of tachyarrhythmia, the closer to the center the rotor is, the higher the dominant frequency tends to be; this is because the center of the rotor has a relatively higher rotation rate than the peripheral portions (Delmar and Anumonwo, 2009).

Phase singularity (PS) refers to the point at which continuous connectivity of the excitation phase in myocardial tissue is not defined. When reentrant waves are generated in myocardial tissue, the center of rotation has PS, which indicates a topological defect in the rotor (Clayton et al., 2006). Accordingly, PS can be used as an indicator of the number of rotors and the complexity of the vortex pattern in reentrant waves (Hayward et al., 2009). Moreover, a line connecting phase singularities in three-dimensional myocardial tissue is called the filament of PS (Clayton et al., 2006). Many experimental results suggest that filament formation, fragmentation, and extinction are closely related to the break-up of reentrant waves (Biktashev et al., 1994; Fenton and Karma, 2002; Pathmanathan and Gray, 2015).

It is difficult to directly measure the APD, dominant frequency, PS, and filament from the heart, but they can be inversely derived from time-varying images obtained using optical mapping techniques. In their experimental study, Knollmann et al. (2001) recorded the action potential from an intact mouse heart using monophasic action potential (MAP) recording techniques. They fixed the electrode to the surface of the heart to record the MAP. Furthermore, even though the PS and filament cannot be detected directly from the heart, these can be detected and counted using heart images. Umapathy et al. (2010) successfully detected PS from cardiac fibrillation using phase mapping techniques. Ten Tusscher et al. (2009) examined the vortex filament using a heart model based on experimental data.

Through the APD, the electrophysiological state of myocardial cells can be predicted, and contractility of the myocardial filament can also be indirectly estimated. We succeeded in predicting cardiac contractility in response to changes in the myocardial APD through computer simulations in our previous studies (Imaniastuti et al., 2014; Jeong and Lim, 2018a, b). However, the myocardial APD alone cannot provide information related to the complicated electrical patterns that occur throughout the heart. The distributions of the dominant frequencies, phase singularities, and filaments do not provide quantitative information at the cellular level, such as the APD, but they do provide information regarding the location and distribution of the rotor of reentrant waves.

These electrophysiological parameters are used to quantify the instability of the electrical patterns of the heart by assuming that they are immediately correlated with cardiac contractility (Hu et al., 2013). However, no studies have directly analyzed the correlations between these electrophysiological quantitative indicators and cardiac contractility. Therefore, we aim to identify the individual and integrative correlations between the electrical parameters such as the APD, dominant frequency, PS, filament and mechanical response of the heart during tachyarrhythmia by deriving the applicable single- and multivariable regression equations. The purpose of this study is to investigate the electrophysiological parameters affecting cardiac contractility during tachyarrhythmia.

## Materials and Methods

### Electromechanical Model of the Cardiac Excitation–Contraction Mechanism

**FIGURE 1:**
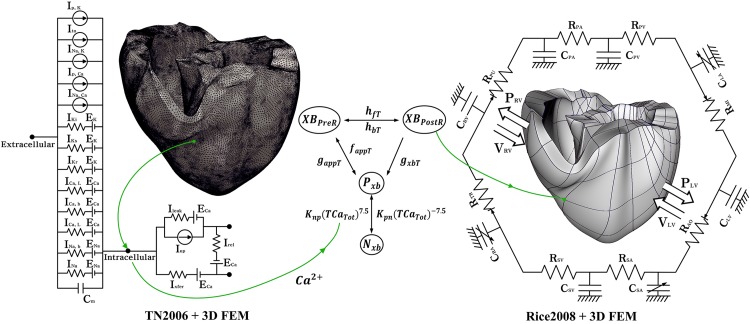
Schematic of the electromechanical model with implementation of one-way coupling in cardiac excitation–contraction mechanism. The left side of the circuit diagram is a human electrophysiological ventricular model, which consists of 619,360 nodes and 3,439,590 tetrahedral elements. The electrical components of the schematic comprise the current, pump, and ion exchanger from Ten Tusscher et al. (2009), which emulate the cell membrane for ion transport and SR within cardiac cells. “I” represents ion currents, and “E” is the equilibrium potential of each ion. The right side is a human mechanical ventricular model, which consists of 14,720 nodes and 6,210 hexahedral elements. The mechanical components represent the excitation–contraction mechanism through the cross-bridge formation of myofilaments suggested by Rice et al. (2008). *N*_*xb*_ and *P*_*xb*_ are non-permissive and permissive confirmations of regulatory proteins, respectively. XB_*PreR*_ and XB_*PostR*_ are the pro-rotated and post-rotated states of the myosin head, respectively. *g*_*xbT*_ is the ATP-consuming detachment transition rate. *h*_*fT*_ and *h*_*bT*_ are the forward and backward transition rates, respectively. *f*_*aapT*_ is the cross-bridge attachment rate of the transition to the first strongly bound state, and g_*aapT*_ is the reverse rate. *K*_*np*_ and *K*_*pn*_ are transition rates. *K_*np*_(TCa_*Tot*_)^7.5^* is the forward rate of the non-permissive-to-permissive transition. *K_*pn*_(TCa_*Tot*_)^– 7.5^* is the backward rate of the permissive-to-non-permissive transition. The electromechanical model is coupled with a circulatory model using the coupling method of Gurev et al. (2011). “R” and “C” represent the resistance and compliance of the cardiac circulatory system, respectively. (For more details, see the text).

To simulate mechanical contraction according to various electrical patterns of ventricular arrhythmia, we used a human ventricular model with two dynamic characteristics, electrical conduction and mechanical contraction (Figure 1). The three-dimensional human ventricular model with electrical conduction characteristics consists of 619,360 nodes and 3,439,590 tetrahedral elements. We have provided a detailed explanation of the modeling of the heart geometry in the Supplementary Material. The model also includes a lumped circulatory circuit that can simulate the ion exchange mechanism through myocardial cell membranes using the validated ventricular cell model developed by Ten Tusscher (2004) and Ten Tusscher and Panfilov (2006) and three-dimensional finite element analysis. In the three-dimensional human ventricular model, the conduction phenomenon of the action potential is expressed using the following equation:

(1) $$\frac{d\,V_{m}}{d\,t}=-\frac{I_{i\,o\,n}+I_{s\,t\,i\,m}}{C_{m}}+\frac{1}{\rho_{x}\,S_{x}\,C_{m}}\,\frac{\partial^{2}V}{\partial^{2}x^{2}}+\frac{1}{\rho_{y}\,S_{y}\,C_{m}}\,\frac{\partial^{2}V}{\partial^{2}y^{2}}+\frac{1}{\rho_{z}\,S_{z}\,C_{m}}\,\frac{\partial^{2}V}{\partial^{2}z^{2}}$$

Here, *V*_*m*_ is the membrane voltage of a myocardial cell and t represents time. *I*_*ion*_ is the sum of the transmembrane currents (Equation 2), *I*_*stim*_ is the current generated by an external stimulus, and *C*_*m*_ is the capacitance of the cell membrane. ρ and *S* are the cellular resistance and ratio of the volume to the surface in every direction, respectively.

(2) $$I_{i\,o\,n}=I_{N\,a}+I_{K\,i}+I_{t\,o}+I_{K\,r}+I_{K\,s}+I_{C\,a,L}+I_{N\,a,C\,a}+I_{N\,a,K}+I_{p,C\,a}+I_{p,K}+I_{C\,a,b}+I_{N\,a,b}$$

*I*_*Na*_ refers to the current of Na^+^ ions, *I*_*Ki*_ is the inward rectifier *K*^+^ current, and *I*_*to*_ is the transient outward *K*^+^ current. *I*_*Kr*_ and *I*_*Ks*_ are the rapid delayed rectifier *K*^+^ current and slow delayed rectifier *K*^+^ current, respectively. *I*_*Ca, L*_ represents the L-type inward Ca^2+^ current, *I*_*Na, Ca*_ is the Na^+^–Ca^2+^ exchange current, and *I*_*Na, K*_ is the Na^+^–K^+^ exchange current. *I*_*p, Ca*_ and *I*_*p, K*_ refer to the pump currents of Ca^2+^ and *K*^2+^, and *I*_*Ca, b*_ and *I*_*Na, b*_ are the background currents of Ca^2+^ and K^+^, respectively. *E*_*K*_, *E*_*Ca*_, and *E*_*Na*_ denote the equilibrium potentials of *K*^+^, Ca^2+^, and Na^+^, respectively.

The three-dimensional human ventricular model with mechanical contraction is composed of 14,720 nodes and 230 hexahedral elements based on Hermite, which can represent the natural ventricular surface curve. Depolarization of each myocyte occurs when electrical wave propagates on the heart and activates the calcium channel to release calcium from the sarcoplasmic reticulum into the cytosol. This released calcium binds to the troponin C, and then cause the cross-bridge contraction due to the sliding of the myofilaments. This progress is shown in Figure 1. *N*_*xb*_ and *P*_*xb*_ are non-permissive and permissive confirmations of regulatory proteins, respectively. XB_*PreR*_ and XB_*PostR*_ are the pro-rotated and post-rotated states of the myosin head, respectively. *g*_*xbT*_ is the ATP-consuming detachment transition rate. *h*_*fT*_ and *h*_*bT*_ are the forward and backward transition rates, respectively. *f*_*aapT*_ is the cross-bridge attachment rate of the transition to the first strongly bound state, and *g*_*aapT*_ is the reverse rate. *K*_*np*_ and *K*_*pn*_ are transition rates. *K_*np*_(TCa_*Tot*_)^7^.^5^* is the forward rate of the non-permissive-to-permissive transition. *K_*pn*_(TCa_*Tot*_)^–7^.^5^* is the backward rate of the permissive-to-non-permissive transition.

The mechanical model simulates contractions of myocardial cells through these calcium dynamics by expanding the cross-bridge model developed by Rice et al. (2008) to the three-dimensional human heart model and subjecting it to three-dimensional finite element methods. For excitation–contraction coupling using calcium, we used the transient calcium information extracted from electrophysiological simulations using calcium dynamics equation as the input for the mechanical simulation (Ten Tusscher and Panfilov, 2006; Rice et al., 2008).

(3) $$\frac{{\mathrm{dCa}}_{\text{itotal}}}{\mathrm{dt}}=-\frac{I_{\mathrm{Ca},L}+I_{b,\mathrm{Ca}}+I_{p,\mathrm{Ca}}-2\,I_{\mathrm{Na},\mathrm{Ca}}}{2\,V_{\text{C}}\,F}+I_{\text{leak}}-I_{\text{up}}+I_{\text{rel}}$$

(4) $$\frac{{\mathrm{dCa}}_{\text{srtotal}}}{\mathrm{dt}}=\frac{V_{\text{c}}}{V_{\text{SR}}}\,\left(-I_{\text{leak}}+I_{\text{up}}-I_{\text{rel}}\right)$$

In these equations, Ca_*itotal*_ is the total amount of calcium in the cytoplasm, and Ca_*srtotal*_ is the total amount of calcium in the sarcoplasmic reticulum (SR). I_*rel*_ is the calcium current released from the junctional SR (JSR), and *I*_*leak*_ is the leakage calcium current of the JSR. *I*_*up*_ is the absorbed calcium current in the network SR (NSR), and *I*_*xfer*_ is the diffusible calcium current between the dyadic subspace and bulk cytoplasm.

Mathematical description of cardiac tissue contraction is based on continuum mechanics (Guccione et al., 1995; Usyk et al., 2002; Gurev et al., 2011), where myocardium is assumed to be hyper-elastic, nearly incompressible material and to have the passive mechanical properties. The passive mechanical properties are defined by an exponential strain function (W).

(5) $$W=\frac{\text{C}}{2}\,\left(e^{Q}-1\right)$$

(6) $$\text{Q}=b_{\text{f}}\,E_{\text{ff}}^{2}+b_{\text{t}}\,\left(E_{\text{rr}}^{2}+E_{\text{cc}}^{2}+2\,E_{\text{rc}}^{2}\right)+2\,b_{\text{fs}}\,\left(E_{\text{fr}}^{2}+E_{\text{fc}}^{2}\right)$$

(7) $$E_{\alpha \,\beta }=\frac{1}{2}\,\left(\frac{\partial x^{k}}{\partial v^{\alpha }}\,\frac{\partial x^{k}}{\partial v^{\beta }}-\delta_{\alpha \,\beta }\right)$$

where *C* is the material constant and set to 2 kPa. The form of *Q* has decided the material that is transversely isotropic with respect to the muscle fiber axis (Guccione and McCulloch, 1991). *b*_*f*_ is 8, *b*_*t*_ is 2 and *b*_*fs*_ is 4, which are determined with the orthotropic electrical conductivity and passive mechanical properties of the myocardium by the laminar sheet-nominal direction and fiber orientation information. *E*_*ij*_ is the Langian Green’s strain, which is referred to the local fiber coordinate system; *E*_*ff*_ is fiber strain, *E*_*rr*_ is the cross-fiber in-plane strain, *E*_*cc*_ is the radial strain, *E*_*rc*_, *E*_*fr*_, and *E*_*fc*_ are shear in the transverse plane, fiber-cross fiber, and fiber-radial coordinate planes, respectively (Guccione et al., 1995).

To simulate the hemodynamic response through the contraction of ventricles, we combined the finite-element electromechanical ventricular model with a circulatory model using the coupling method proposed by Gurev et al. (2011). The circulatory model is based on the cardiovascular model developed by Kerckhoffs et al. (2007). The human cardiovascular model consists of a lumped hemodynamic model, as shown on the right-hand side of Figure 1. In Figure 1, *C*_*PA*_ and *R*_*PA*_ are the compliance and resistance of the pulmonary artery, and *C*_*PV*_ and *R*_*PV*_ are the compliance and resistance of the pulmonary vein, respectively. *C*_*LA*_ and *R*_*MI*_ are respectively the compliance of the left atrium and resistance of the mitral valve; *C*_*LV*_ and *R*_*AO*_ are respectively the compliance of the left ventricle and resistance of the aortic valve; *C*_*SA*_ and *R*_*SA*_ are the compliance and resistance of the systemic artery, respectively; *C*_*SV*_ and *C*_*SV*_ are respectively the compliance and resistance of the systemic vein; *C*_*RA*_ and *R*_*TR*_ are the compliance of the right atrium and resistance of the tricuspid valve, and *C*_*RV*_ and *R*_*PU*_ are the compliance of the right ventricle and resistance of the pulmonary valve, respectively. *P*_*RV*_ and *V*_*RV*_ are the pressure and volume of the right ventricle, and *P*_*LV*_ and *V*_*LV*_ are the pressure and volume of the left ventricle, respectively.

Therefore, we could calculate the hemodynamic response at the tissue level according to the electrophysiological results of ventricular tachyarrhythmia simulations. From the cardiovascular model, the ventricular pressure was calculated using Equations 8–11.

(8) $$Pressure=C^{-1}\left(t\right)(V-V_{\text{rest}}\left(t\right)$$

(9) $$\Delta \,\vec{V}=C\cdot \vec{P}=\left[\begin{array}{c} \Delta \,V_{\text{L}}\\ \Delta \,V_{\text{R}} \end{array}\right]=\left[\begin{array}{cc} C_{\text{LL}} & C_{\text{LR}}\,\left(p_{\text{L}}\right)\\ C_{\text{RL}}\,\left(p_{\text{R}}\right) & C_{\text{RR}} \end{array}\right]\,\left[\begin{array}{c} p_{\text{L}}\\ p_{\text{R}} \end{array}\right]$$

(10) $$C=y_{\text{v}}\,\left(C_{\text{max}}-C_{\text{min}}\right)+C_{\text{min}}$$

(11) $$V_{\text{rest}}=\left(1-y_{\text{v}}\right)*\left[\begin{array}{cc} V_{L,\mathrm{rest},d} & -V_{L,\mathrm{rest},s}\\ V_{R,\mathrm{rest},d} & -V_{R,\mathrm{rest},s} \end{array}\right]+\left[\begin{array}{c} V_{L,\mathrm{rest},s}\\ V_{R,\mathrm{rest},d} \end{array}\right]$$

Here, *C* is the time-varying compliance matrix; *C*_*LL*_ and *C*_*RR*_ are the time-varying compliance matrices of the left and right ventricles, respectively. These were calculated from the compliance under the active state (*C*_*Max*_) and passive state (*C*_*Min*_) of ventricles (Equation 9). *y*_*v*_ is the activation function of the ventricles, and *V* denotes the volume; *V*_*rest*_ is the volume when the pressure of the ventricle is 0; *V*_*L,rest,d*_ and *V*_*L,rest,s*_ are the left ventricular volumes in the systolic and diastolic periods, respectively, and *V*_*R,rest,d*_ and *V*_*R,rest,s*_ are the right ventricular volumes in the systolic and diastolic periods, respectively.

### Simulation Protocols for Generating Reentrant Waves

**FIGURE 2:**
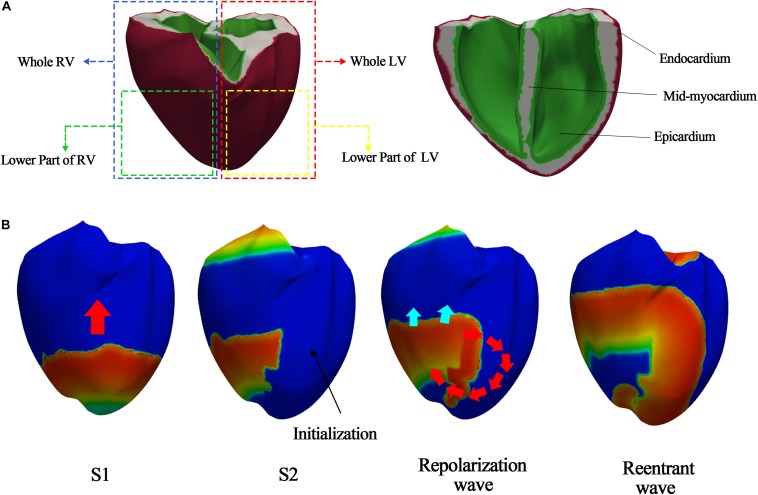
S1–S2 protocols according to S2 stimulus parts. **(A)** S2 stimulus parts to generate the various ventricular tachyarrhythmia conditions; **(B)** example of reentry generated when stimulus S2 is given to lower part of left ventricle.

To observe the electrical patterns and contractility using sustained reentrant waves, we performed simulations involving reentry generation and reentry sustaining. For reentry generation, we used the S1–S2 protocols to generate reentrant waves in the three-dimensional human ventricular tissue model under a low conduction velocity (20 cm/s), because reentry is easily generated under a low conduction velocity. First, stimulus S1 was activated thrice every 600 ms, and stimulus S2 was activated when the wave tail of the last S1 stimulus reached the middle portion of the ventricular model (Figure 2B). The reentry generation simulation was performed for 10 s. We saved the state of the myocardial cells at the last moment of reentry and used it as the input for the reentry maintenance simulation to observe sustained reentry under a normal conduction velocity (68.5 cm/s). The conduction velocity was calculated by dividing the height of the ventricular model by the time it took for the propagating waves to move from the apex to the top of the ventricle. The sustained reentry simulation under the normal conduction velocity ran for up to 20 s for observation of the electrical and mechanical properties at the moment when the reentrant waves reached a steady state.

We used two methods to generate various electrical patterns caused by ventricular tachyarrhythmia. First, we changed the electrical conductance of the *I*_*Ks*_ channel (*g*_*Ks*_), the protein channel that has the greatest effect on the electrical activity of the heterogeneous ventricular tissue. Even though the electrical conductances of the *I*_*Na*_ and *I*_*Ki*_ channels are higher than that of *I*_*Ks*_ channel (*g*_*Ks*_), they are values that do not consider the heterogeneity of ventricular tissue according to the Ten Tusscher ion model. The electrical conductances of only *I*_*to*_ and *I*_*Ks*_ consider the heterogeneity of ventricular tissue. As the electrical conductance of *I*_*to*_ affects phase 0 of the action potential generation, we doubled the value of *g*_*Ks*_ and then increased it to 4-, 6-, 8-, 10-, 20-, 30-, 40-, 60-, 80-, and 100-fold from the normal value (*g*_*Ks*_ = 0.392^∗^1.3 mS/μF). This was done for creating a regression model that can cover a wide range from a general to extreme situation. Second, we varied stimulus S2 of the S1–S2 protocols in the reentry generation simulation to generate various electrical patterns. S2 affected the entire left ventricle, lower parts of the left ventricle, entire right ventricle, or lower parts of the right ventricle (Figure 2A).

### Quantification of Electrical and Mechanical Characteristics During the Ventricular Tachyarrhythmia

The following four values were used to quantify the various electrical patterns during the occurrence of tachyarrhythmia.

1. APD: The APD was obtained by measuring the time it took from depolarization to 90% repolarization of the myocardial cells in one cycle. Supplementary Figure S1B shows the action potential shape from 16,000 to 18,000 ms during sustained reentry. The APD during tachyarrhythmia was calculated over 10 s at the same position (center) in each case to consider the dramatically changed electrical excitation. If the action potential cycles were generated 10 times during tachyarrhythmia, the APD was also measured 10 times. Finally, the APD during reentry was averaged and used for regression analysis.

Dominant frequency: We performed frequency analysis using the membrane potential signals obtained at each node of the ventricular model during reentry. The frequency of the highest power band was defined as the dominant frequency (Ng et al., 2006). Frequency analysis was performed at a sampling frequency of 0.01 Hz using the fast Fourier transform function in MATLAB. The dominant frequency value in the case of tachyarrhythmia was the mean value of the dominant frequency calculated at all nodes.

1. PS: To detect the PS of the reentrant waves, we converted the membrane potential information obtained at each node of the ventricular model into phase information in the phase variable state-space using Equation 12 (Iyer and Gray, 2001).

(12) $$\theta \,\left(x,y,z,t\right)=a\,r\,c\,t\,a\,n\,2\,\left(\frac{V\,\left(x,y,z,t+\tau \right)-V_{\text{mean}}}{V\,\left(x,y,z,t\right)-V_{\text{mean}}}\right)$$

**FIGURE 3:**
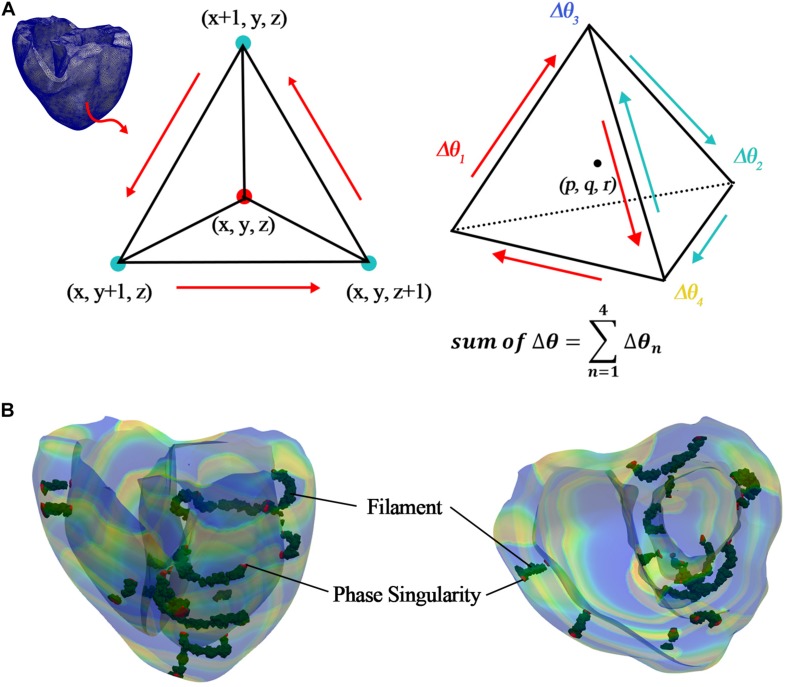
Detection of PS and filament. **(A)** Detecting PS and filament in tetrahedral elements; **(B)** detected PS (red elements) and filament (green elements) in ventricular model, which consists of tetrahedral elements.

Here, arctan2 is the arctan function that considers the quadrant and returns a phase value between –π and + π. τ is the time delay for calculating the phase of each node and was set to 10 ms in this study. This is equivalent to the time resolution of the three-dimensional result files obtained through the electrophysiological simulation and was set considering computational efficiency because we used a high-resolution ventricular model consisting of 619,360 nodes. *V*_*mean*_ refers to the ideal origin mentioned in the prior study of Iyer and Gray (2001). In this study, we used the mean value of the membrane potential at time *t* when reentry occurred. The PS is the point at which the connectivity of the excitation wave’s phase is undefined. Therefore, the point at which the sum of the phase differences in the vicinity became ±2π was detected, and it was considered to be a PS (Figure 3A and Equation 13). The phase difference at each point was calculated using Equation 14, and it showed a wide distribution range [−π, π).

(13) $$\mathrm{Sum}\,\mathrm{of}\,\Delta \,\theta =\sum_{n=1}^{4}\Delta \,\theta_{n}=\left\{ \begin{array}{c} \pm 2\,\pi ,P\,S\,p\,o\,i\,n\,t\\ 0,O\,t\,h\,e\,r\,w\,i\,s\,e \end{array}\right.$$

(14) $$P\,h\,a\,s\,e\,D\,i\,f\,f\,e\,r\,e\,n\,c\,e\,\left(P\,D\right)=\left\{ \begin{array}{c} \theta_{1}-\theta_{2},\left|\mathrm{PD}\right|\leq \pi \\ \theta_{1}-\theta_{2}-2\,\pi ,\left|\mathrm{PD}\right|>\pi ,P\,D>0\\ \theta_{1}-\theta_{2}+2\,\pi ,\left|\mathrm{PD}\right|>\pi ,P\,D<0 \end{array}\right.$$

Then, we detected the PS points in the ventricular geometry, as shown in Figure 3B. To quantify the PSs, we counted the number of PSs, which were detected in the ventricles during reentry according to time, and averaged them. Finally, the average number of PSs during reentry was used for regression analysis.

1. Filaments: Filaments were detected by applying the method proposed by Fenton and Karma, which detects filaments in a three-dimensional cube (Fenton and Karma, 2002). Then, we found a point satisfying the iso-potential condition both temporally (Equation 15) and spatially (Equation 16) simultaneously and we considered it to represent the filaments.

(15) $$\frac{{\mathrm{dV}}_{\text{m}}}{\mathrm{dt}}=0$$

(16) $$V_{\text{m}}^{\text{n}}=V_{\text{m}}^{n+1}=V_{\text{iso}}$$

In the equations, *V*_*iso*_ was set between −75 and −10 mV, depending on the mean range of the membrane potentials of the PSs detected at the ventricular tissue surface. This considers the characteristic that filaments internally connect the PSs to the surface. Furthermore, the points satisfying the spatial iso-potential condition were regarded as the points where changes in the potentials at the centers (p, q, and r) of tetrahedral elements were the same (Equations 17 and 18, and Figure 3A).

(17) $$V_{\text{m}}^{\text{n}}=p\cdot V_{x,y,z}^{\text{n}}+q\cdot V_{x+1,y,z}^{\text{n}}+r\cdot V_{x,y+1,z}^{\text{n}}+\left(1-p-q-r\right)\cdot V_{x,y,z+1}^{\text{n}}$$

(18) $$V_{\text{m}}^{n+1}=p\cdot V_{x,y,a}^{n+1}+q\cdot V_{x+1,y,z}^{n+1}+r\cdot V_{x,y+1,z}^{n+1}+\left(1-p-q-r\right)\cdot V_{x,y,z+1}^{n+1}$$

Here *p*, *q*, and *r* all have values between (0 and 1). To determine the center point of the tetrahedral elements, we set the values of *p*, *q*, and *r* to 0.5. Then, we could detect the filaments in the ventricular geometry, as shown in Figure 3B. To quantify the filaments, we counted the number of filaments, which were detected in the ventricles during reentry according to time, and averaged them. Finally, the average number of filaments during reentry was used for regression analysis.

We used the stroke volume (SV) and amplitude of the tension (ampTens) as quantitative values to evaluate the contraction efficiency according to various electrical patterns.

1. SV: The volume of the left ventricle was measured during reentry using the electromechanical model coupled with a cardiovascular model. We defined a meaningful period, which is the time when blood actually flows in and out of the left ventricle during tachyarrhythmia (Supplementary Figure S4). During the meaningful period, we measured the end-diastolic volume, which is the volume right before when the ventricular volume maximally increased and then decreased, and the end-systolic volume, which is the volume right before when the ventricular volume minimally decreased and then increased. The SV values were obtained by taking the difference between the volumes at the end of the diastole and at the end of the systole during this period. Then, we used the average SV value as the quantitative value of contractility during tachyarrhythmia.
2. ampTens: We measured the standard deviation of the myocardial tension according to the various electrical patterns generated by tachyarrhythmia using the electromechanical model. The contraction and relaxation of the myocardium were determined as the quantitative values of contractility during tachyarrhythmia. Therefore, the amplitude of the tension was used as the value representing contractility. For this reason, we calculated the mean value of the standard deviation of the tension obtained at all nodes of the human ventricular model.

### Determination of Electrical Parameters Influencing Mechanical Contractility

We performed the regression analysis using the “IBM SPSS statistics 25” program to identify the electrical parameters affecting mechanical contractility during tachyarrhythmia. We used four electrical parameters as the predictors for regression analysis, namely the APD, dominant frequency, PS, and filaments. As the unit of each electrical value is different, we standardized the electrical parameters so that the mean was zero and the variance was one. The SV and ampTens were set as the dependent variables. The dependent variables were transformed such that the mean and variance of error were zero and one, respectively. Then, we created regression modes for predicting the SV and ampTens from the electrical parameters. Using the “enter” regression as the estimation method, it was possible to confirm the influence of specific independent variables under the control of other independent variables. To test the fitness of the regression model, we performed the analysis of variance (ANOVA) tests.

First, we carried out correlation analyses to confirm the individual relationships between the variables. Then, single regression analyses were performed to identify the individual influences of the electrical parameters on cardiac contractility. Furthermore, we conducted multiple regression analyses to determine the electrical parameters influencing mechanical contraction when all the electrical variables were present concurrently. We performed collinearity tests by calculating the variance inflation factor (VIF) and confirmed its potential influence on the models. Then, we identified and corrected the correlations between predictors that could negatively impact the results of the multiple regression analysis based on the VIF values. After eliminating the predictors with collinearity, we derived multiple regression models and compared the effects of the independent electrical variables on cardiac dynamics.

## Results

### Results of Electromechanical Simulation

**FIGURE 4:**
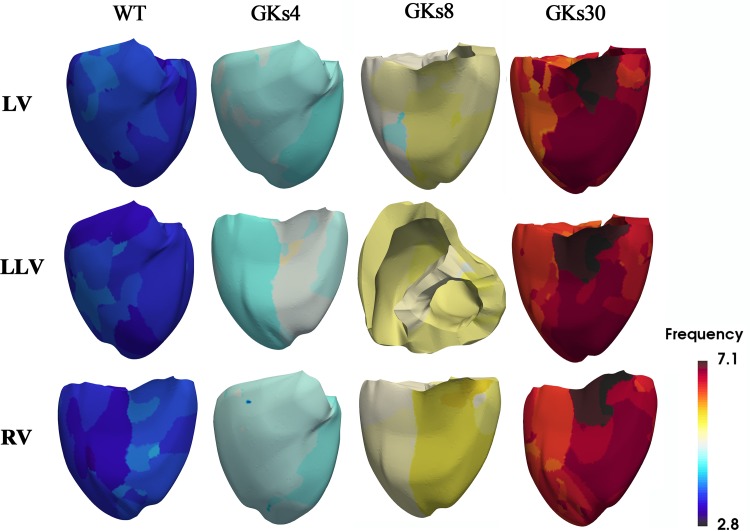
Dominant frequency contours: WT, wild type; GKs4, GKs8, and GKs30, conditions under which the conductance of *I*_*Ks*_ channel doubled and increased to 4-, 8-, and 30-fold; LV, LLV, and RV are the conditions under which stimulus S2 was given to the whole left ventricle, lower part of the left ventricle, and the whole right ventricle, respectively.

**FIGURE 5:**
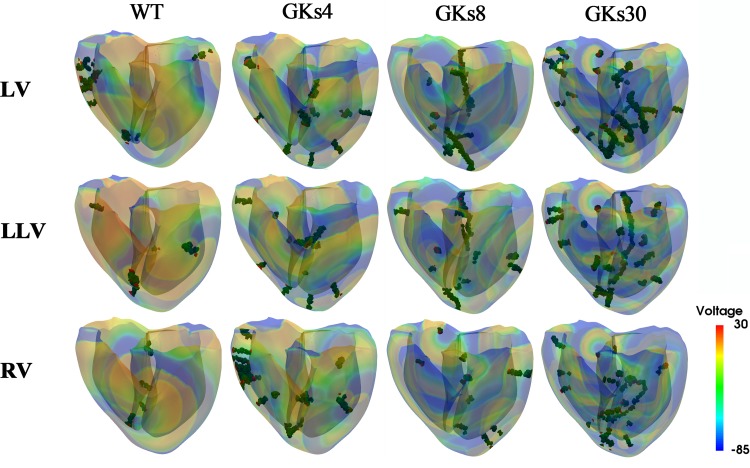
Membrane voltage contours with phase singularities and filaments: WT, wild type; GKs4, GKs8, GKs30, the conditions that the conductance of *I*_*Ks*_ channel increased doubled, 4-, 8-, and 30-fold, respectively; LV, LLV, and RV, the conditions that stimulus S2 was given to whole left ventricle, the lower part of left ventricle, and the whole right ventricle, respectively.

We implemented 48 types of tachyarrhythmia signals by changing the conduction characteristics of the *I*_*Ks*_ channel, which has the greatest effect on the electrical activity of the ventricular tissue and applying four types of reentrant wave generation methods. From the electrophysiological simulation, we obtained the electrical instability parameters under the 48 types of tachyarrhythmia conditions. The following dataset comprising the electrical instability parameters was obtained through the simulation: The APD is 131.02 ± 50.47 ms (Supplementary Figure S1); the average of the local dominant frequencies is 5.59 ± 1.15 Hz (Figure 4 and Supplementary Figure S2); the average number of PSs is 49.98 ± 24.49, and the average number of filaments is 12,401.1 ± 7,902.9 (Figure 5 and Supplementary Figure S3). The dataset for the mechanical parameters obtained through the electromechanical simulation is as follows: The average SV is 0.38 ± 0.56 mL (Supplementary Figure S4), and the average ampTens is 0.41 ± 0.38 kPa (Supplementary Figure S5).

### Correlations Between Individual Parameters

**FIGURE 6:**
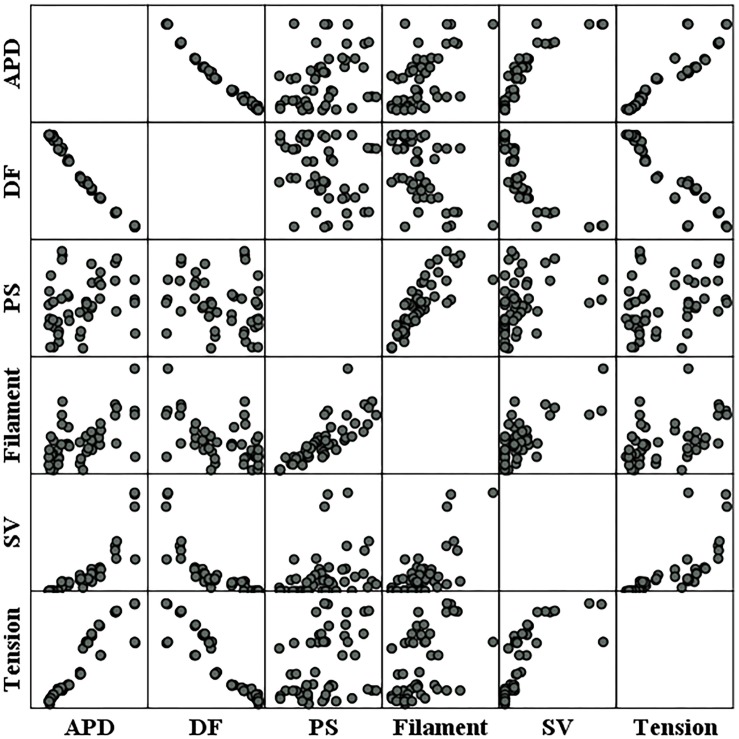
Scatter plots of electrical parameters and mechanical parameters. APD, action potential duration; DF, dominant frequency; PS, the number of phase singularities; Filament, the number of filaments; SV, stroke volume.

Figure 6 shows the individual correlations between the electrical and mechanical parameters. Among the parameters representing electrical phenomena, the linear relationship between the APD and SV was the strongest (*R* = 0.859, *p*-value < 0.05). The dominant frequency has a strong negative linear correlation with the SV, and the SV decreases with the dominant frequency (*R* = −0.809, *p*-value < 0.05). The next strongest correlation is between the filaments and SV (*R* = 0.713, *p*-value < 0.05). As for the PS, its correlations with the SV is lower compared to the other electrical parameters (*R* = 0.305, *p*-value < 0.05; Table 1).

**TABLE 1:** Correlation coefficients.

		SV	Tension	APD	DF	PS	Filament
Pearson correlation coefficient (R)	SV	1.000	0.887	0.859	−0.809	0.305	0.713
	Tension	0.887	1.000	0.930	−0.907	0.146	0.507
	APD	0.859	0.930	1.000	−0.991	0.284	0.577
	DF	−0.809	−0.907	−0.991	1.000	−0.268	−0.533
	PS	0.305	0.146	0.284	−0.268	1.000	0.795
	Filament	0.713	0.507	0.577	−0.533	0.795	1.000
*p*-value	SV	.	0.000	0.000	0.000	0.018	0.000
	Tension	0.000	.	0.000	0.000	0.161	0.000
	APD	0.000	0.000	.	0.000	0.025	0.000
	DF	0.000	0.000	0.000	.	0.033	0.000
	PS	0.018	0.161	0.025	0.033	.	0.000
	Filament	0.000	0.000	0.000	0.000	0.000	.
*N*	SV	48	48	48	48	48	48
	Tension	48	48	48	48	48	48
	APD	48	48	48	48	48	48
	DF	48	48	48	48	48	48
	PS	48	48	48	48	48	48
	Filament	48	48	48	48	48	48

*SV, stroke volume; tension, standard deviation of tension; APD, action potential duration; DF, dominant frequency; PS, the number of phase singularities; Filament, the number of filaments; N, the quantity of data.*

A single regression model has derived to observe the sensitivity of the SV according to changes in the electrical parameters (Table 2). Variations in the SV according to unit changes in the electrical parameters are the highest at the dominant frequency (unstandardized B coefficient = −0.393, *p*-value < 0.05) and lowest at the filaments (unstandardized B coefficient = 5.059E-5, *p*-value < 0.05). The sensitivity of the SV according to one unit change in the APD is 0.010 (*p*-value < 0.05), and that of the SV according to one unit change in the PS is 0.007 (*p*-value < 0.05).

**TABLE 2:** Single-variable regression models for predicting SV.

Model		Unstandardized coefficient		Standardized coefficient	*t*	Sig. *t* (*p*-value)	Model summary		
						
		*B*	*SE*	Beta			*R*^2^	*F*	Sig. *F*
A1	(Intercept)	−0.868	0.117		−7.390	0.000	0.738	129.7	0.000
	APD	0.010	0.001	0.859	11.389	0.000			
A2	(Intercept)	2.583	0.240		10.573	0.000	0.655	87.4	0.000
	DF	−0.393	0.042	−0.809	−9.351	0.000			
A3	(Intercept)	0.034	0.179		0.188	0.852	0.093	4.7	0.035
	PS	0.007	0.003	0.305	2.170	0.035			
A4	(Intercept)	−0.245	0.107		−2.282	0.027	0.509	47.6	0.000
	Filament	5.059E-5	0.000	0.713	6.902	0.000			

*APD, action potential duration; DF, dominant frequency; PS, the number of phase singularities; Filament, the number of filaments; B, beta coefficient; SE, standard error; t, T-statistics; sig.t, significant value of T-statistics; R^2^, coefficient of determination; F, F-statistics; sig. F; significant value of F-statistics. Model A1 is used for the APD, Model A2 is used for the dominant frequency, Model A3 is used for phase singularities, and Model A4 is used for length of filaments considered as independent variables, respectively.*

Standardized regression coefficients are used to relatively compare the individual influences of the electrical parameters on the SV using different units of measure. The APD has the statistically strongest influence on the SV (standardized beta coefficient = 0.859, *p*-value < 0.05), and the effect of the PS is the weakest (standardized beta coefficient = 0.305, *p*-value < 0.05). The second most influential parameter is the dominant frequency (standardized beta coefficient = −0.809, *p*-value < 0.05). The third most influential parameter is the filament (standardized beta coefficient = 0.713, *p*-value < 0.05).

It is possible to statistically predict the SV using a single regression equation relating to each electrical parameter and the SV. Among the four electrical parameters, the accuracy (*R*^2^) in predicting the SV is the highest at 73.8% (*p*-value < 0.05) using the APD, which has the highest correlation with the SV. The standard error (SE) between the stochastically predicted SV using the single regression equation and predicted SV through deterministic simulation is ±0.290 mL. The accuracy of predicting the SV based on the dominant frequency is 65.5% (*p*-value < 0.05), and that using the filament is 50.9% (*p*-value < 0.05). The accuracy of stochastically estimating the SV using the PS is statistically the lowest, at 9.3% (*p*-value < 0.05).

Ventricular ejection during tachyarrhythmia is very irregular. Consequently, it is difficult to determine a meaningful ejection period as the severity of tachyarrhythmia increases. Therefore, we used the ampTens in the myocardium to obtain more objective and quantitative values as parameters to estimate mechanical contractility in tachyarrhythmia. Among the electrical parameters, the APD and dominant frequency are more highly correlated with the myocardial ampTens than with the SV during reentry (Figure 4 and Table 1). Similar to the SV, there is a statistically significant positive correlation between the ampTens and APD, which is the highest at −0.930 (*p*-value < 0.05). Furthermore, the myocardial ampTens has a statistically strong negative correlation with the dominant frequency (Pearson correlation coefficient = −0.907, *p*-value < 0.05). The correlation between the filament and ampTens is moderate at 0.507 (*p*-value < 0.05), which is lower than the correlation between the filament and SV. The PS do not have any statistical correlation with the ampTens (*p*-value = 0.161).

Single regression models have derived from the individual relationships between the ampTens and three electrical parameters representing electrical activity, excluding the PS (three parameters having statistically significant relationships with ampTens), are able to determine the effects of the APD, dominant frequency, and filament on myocardial tension (Table 3). The variation in myocardial ampTens according to unit changes in the three electrical parameters is the largest for a unit change in the dominant frequency (unstandardized B coefficient = −0.299, *p*-value < 0.05). The change in the ampTens due to a unit change in the APD is the second largest (unstandardized B coefficient = 0.07, *p*-value < 0.05), and the change in myocardial tension due to a unit change in the filament is the smallest (unstandardized B coefficient = 2.441E-5, *p*-value < 0.05). These results are similar to the variations observed in the SV according to unit changes in each electrical parameter.

**TABLE 3:** Single-variable regression models for predicting the tension amplitude.

Model		Unstandardized coefficient		Standardized coefficient	*t*	Sig. *t* (*p*-value)	Model summary		
						
		*B*	*SE*	Beta			*R*^2^	*F*	Sig. *F*
B1	(Intercept)	−0.510	0.057		−8.931	0.000	0.866	296.8	0.000
	APD	0.007	0.000	0.930	17.227	0.000			
B2	(Intercept)	2.084	0.117		17.791	0.000	0.822	212.9	0.000
	DF	−0.299	0.021	−0.907	−14.590	0.000			
B3	(Intercept)	0.107	0.090		1.188	0.241	0.257	15.90	0.000
	Filament	2.441E-5	0.000	0.507	3.987	0.000			

*APD, action potential duration; DF, dominant frequency; PS, the number of phase singularities; Filament, the number of filaments; B, beta coefficient; SE, standard error; t, T-statics; sig.t, significant value of T-statistics; R^2^, coefficient of determination; F, F-statistics; sig. F; significant value of F-statistics. Model B1 is used for the APD, Model B2 is used for the dominant frequency, and Model B3 is used for the number of filaments as an independent variable*.

We have compared the relative influence of the three electrical parameters on the myocardial ampTens. Statistically, the most influential independent variable is the APD (standardized beta coefficient = 0.930, *p*-value < 0.05), followed by the dominant frequency (standardized beta coefficient = −0.907, *p*-value < 0.05). The relative sensitivity of the ampTens to the APD and dominant frequency is higher than the sensitivity of the SV to these variables. However, the standardized regression coefficient of the filament is 0.507 (*p*-value < 0.05), and the relative sensitivity of the ampTens to the filament is lower than that of the SV.

We have estimated the ampTens through a single regression model derived using the three electrical parameters individually, excluding the PS. The prediction accuracy of the ampTens is the highest at 86.6% (*p*-value < 0.05), obtained from the single regression model using the APD as the predictor. It is possible to predict the ampTens with higher accuracy compared to the SV using the APD. The SE between the ampTens stochastically predicted from the APD and that deterministically predicted from tachyarrhythmia simulations is ±0.141 kPa. Furthermore, the single regression model using the dominant frequency as the predictor of the ampTens has a high prediction accuracy of 82.2% (*p*-value < 0.05), which is higher than the accuracy obtained when predicting the SV using the dominant frequency. However, when the ampTens is stochastically estimated using the filament as the predictor, the accuracy is lower than that of the predicted SV (*R*^2^ = 0.257, *p*-value < 0.05). This is statistically less accurate than the single regression model using either the APD or dominant frequency.

Further, there is a strong negative correlation between the dominant frequency and APD, with a correlation coefficient of −0.991 (*p*-value < 0.05). This correlation is higher than that observed in the relationships between the APD and dominant frequency with the SV and ampTens. Furthermore, there is a statistically strong positive correlation between the PS and filament (*R* = 0.795, *p*-value < 0.05). The correlation coefficient between these parameters is also higher than their correlation coefficients with the SV or ampTens.

### Multivariable Regression Analyses to Discover the Most Influential Electrophysiological Feature for Estimating the Severity of VF

We have performed the following multivariable regression analyses between the electrical parameters (APD, dominant frequency, PS, and filament) and mechanical parameters (stroke volume and ampTens) using 48 types of tachyarrhythmia simulation data: (1) between the four electrical parameters and SV (Model 1); (2) between three electrical parameters and the SV [APD was statistically excluded (Model 2)]; (3) between the four electrical parameters and ampTens (Model 3); (4) between three electrical parameters (APD was statistically excluded) and ampTens (Model 4). Table 4 shows summaries of all regression models.

**TABLE 4:** Regression model summary.

Model	*R*	Adjusted *R*^2^	*SE*	*R*^2^ change	*F*	df1	df2	Sig. *F* (*p*-value)	Durbin–Watson
1	0.949	0.892	0.18438	0.901	97.845	4	43	0.000	1.797
2	0.923	0.841	0.22343	0.851	83.932	3	44	0.000	1.415
3	0.949	0.891	0.12555	0.901	97.290	4	43	0.000	2.001
4	0.930	0.856	0.14467	0.865	93.834	3	44	0.000	1.781

*R, correlation coefficient; R^2^, coefficient of determination; SE, standard error of estimation; F, F-statistics; df, degrees of freedom; Sig. F, significant value of F-statistics*. *In Model 1, all electrical variables were used to predict SV. In Model 2, three electrical variables (APD was excluded) were used to predict SV. In Model 3, all electrical variables were used to predict tension. In Model 4, all electrical variables except APD were used to predict tension*.

Although not all the four electrical parameters have shown a linear correlation with the SV (PS is not linearly related to SV in the correlation analysis, see Table 1), we are able to build a multivariable regression model with a statistical accuracy of 89.2% (Model 1, *p*-value < 0.05) by concurrently considering the four electrical parameters. The mean error between the stochastically predicted SV using Model 1 and the deterministically predicted SV via simulation is ±0.137 mL. When the influences of the four electrical parameters are considered simultaneously, we have observed that the APD has the greatest effect on the SV (standardized beta coefficient = 1.983, *p*-value < 0.05), followed by the dominant frequency (standardized beta coefficient = 1.371, *p*-value < 0.05), filament (standardized beta coefficient = 0.582, *p*-value < 0.05), and PS (standardized beta coefficient = −0.354, *p*-value < 0.05). However, the APD and dominant frequency showed very high multicollinearity (79.032 VIF for APD and 71.137 VIF for the dominant frequency; see Table 5).

**TABLE 5:** Significance of independent variables.

Model		Unstandardized coefficient		Standardized coefficient	*t*	Sig. *t* (*p*-value)	Collinearity statistics	
						
		*B*	*SE*	Beta			Tolerance	VIF
1	(Intercept)	−6.339	1.705		−3.717	0.001		
	APD	0.022	0.005	1.983	4.649	0.000	0.013	79.032
	DF	0.666	0.197	1.371	3.389	0.002	0.014	71.137
	PS	−0.008	0.002	−0.354	−3.972	0.000	0.290	3.450
	Filament	4.128E-5	0.000	0.582	5.238	0.000	0.186	5.362
2	(Intercept)	1.540	0.229		6.735	0.000		
	DF	−0.238	0.035	−0.490	−6.788	0.000	0.650	1.539
	PS	−0.012	0.002	−0.505	−5.021	0.000	0.334	2.992
	Filament	6.053E-5	0.000	0.853	7.451	0.000	0.258	3.881
3	(Intercept)	−2.558	1.161		−2.203	0.033		
	APD	0.013	0.003	1.679	3.927	0.000	0.013	79.032
	DF	0.252	0.134	0.763	1.881	0.067	0.014	71.137
	PS	−0.004	0.001	−0.226	−2.525	0.015	0.290	3.450
	Filament	5.995E-6	0.000	0.124	1.117	0.270	0.186	5.362
4	(Intercept)	1.973	0.148		13.324	0.000		
	DF	−0.268	0.023	−0.813	−11.816	0.000	0.650	1.539
	PS	−0.005	0.001	−0.353	−3.687	0.001	0.334	2.992
	Filament	1.707E-5	0.000	0.354	3.245	0.002	0.258	3.881

*B, beta coefficient; SE, standard error; t, T-statistics; sig. t, significant value of T-statistics; VIF, variance inflation factor; APD, action potential duration; DF, dominant frequency; PS, the number of phase singularities; Filament, the number of filaments. In Model 1, all electrical variables were used to predict SV. In Model 2, three electrical variables (APD was excluded) were used to predict SV. In Model 3, all electrical variables were used to predict tension. In Model 5, electrical variables except APD were used to predict tension*.

To solve the multicollinearity affecting Model 1, we have removed the APD based on statistical results; according to these results, the APD is the electrical activity variable with the highest VIF index. Then, the dominant frequency, PF, and filament were used to determine if the SV has been predictable (Model 2). The accuracy of the multiple regression model derived from Model 2 is 84.1% (*p*-value < 0.05), which is lower than the accuracy of regression Model 1, but the VIF indices of all three variables have decreased to below the maximum permissible value. When considering the three electrical activity variables used in Model 2, the variable with the highest influence on the SV is the filament (standardized beta coefficient = 0.853, *p*-value < 0.05), followed by the PS (standardized beta coefficient = −0.505, *p*-value < 0.05) and dominant frequency (standardized beta coefficient = −0.490, *p*-value < 0.05, Table 5). The mean error between the statistically predicted SV using Model 2 and that measured through deterministic simulation is ±0.159 mL.

When considering the four electrical activity variables at the same time, we are able to predict the ampTens with an accuracy of 89.1% (*p*-value < 0.05). In Model 3, the electrical parameter that had the largest influence on the myocardial ampTens is the APD (standardized beta coefficient = 1.679, *p*-value < 0.05), followed by the PS (standardized beta coefficient = −0.226, *p*-value < 0.05). Of the four electrical activity variables, the dominant frequency and filament do not significantly affect the myocardial ampTens (*p*-values = 0.067 and 0.270, respectively). In this case, the mean error between the stochastically predicted ampTens using Model 3 and the deterministically predicted ampTens through simulation is ±0.089 kPa. However, as discussed for Model 1, the multicollinearity between the APD and dominant frequency used in Model 3 is very high.

We have derived a regression model similar to Model 2 for predicting the myocardial ampTens by excluding the APD as an electrical activity variable for solving the multicollinearity issue between the APD and dominant frequency (Model 4). Accordingly, the VIF indices of all the electrical activity variables used in Model 4 are reduced to below the permissible value, and all three electrical activity variables significantly have affected the myocardial tension (*p*-value of all variables < 0.05). The most influential variable on the ampTens is the dominant frequency (standardized beta coefficient = −0.813), followed by the filament (standardized beta coefficient = 0.354) and PS (standardized beta coefficient = −0.353). In Model 4, the prediction accuracy for the myocardial ampTens is 85.6% (*p*-value < 0.05). The mean error between the stochastically predicted ampTens using Model 5 and deterministically predicted ampTens through simulation is ±0.145 kPa.

## Discussion

In this study, we have identified the most influential electrical parameter on mechanical contraction among the representative electrical parameters, such as the APD, dominant frequency, PSs, and filaments under tachyarrhythmia conditions. Furthermore, we stochastically have identified the individual and integrative correlations between the electrical phenomena and mechanical contractility, which were predicted using deterministic models. We used the electromechanical models of a three-dimensional ventricle developed by our team and simulated the ventricular excitation–contraction phenomenon during tachyarrhythmia. The main findings of this study are as follows.

(1) We successfully have simulated the electrical excitation-mechanical contraction of the ventricles under a total of 48 different types of tachyarrhythmia, which are induced through a cell membrane model with 12 different action potentials and four reentrant wave generation methods.
(2) The APD, dominant frequency, and filament, excluding the PS, present statistically significant correlations with the SV the myocardial ampTens (Table 1). Among them, the APD has the greatest effect on the two dependent variables (changes in SV and myocardial ampTens), which represent ventricular ejection (standardized beta coefficient: 0.859 of SV, 0.930 of ampTens; Tables 2, 3).
(3) Multicollinearity between the APD and dominant frequency is observed in the multiple regression models. For this reason, we have rederived the multiple regression models to consider only three electrical parameters (dominant frequency, PS, and filament) based on statistical results. The dominant frequency, PS, and filament have statistically significant correlations with the SV and myocardial ampTens (Table 4). In particular, the filaments produce the highest change in the SV (standardized beta coefficient: 0.853), and the dominant frequency has the largest effect on the myocardial ampTens (Standardized beta coefficient: −0.813; Table 5).

Changes in electrical conductivity in the potassium channel shorten the APD of myocardial cells. When the cellular depolarization period is shorter than that of a normal cell, the opening time of the voltage-dependent L-type calcium channel is also shortened. Accordingly, the intracellular calcium concentration decreases, and the cross-bridge formation rate of the myocardial fibers that depends on calcium dynamics decreases, causing a reduction in the contractility of the myocardial cells. Reduced contractility in cardiomyocyte causes decreases in left ventricular ejection and the SV. The complexity of the reentrant wave during tachyarrhythmia negatively affects the ventricular rate. When spiral-wave break-up occurs, multiple reentrant waves (chaotic reentrant waves) are formed, and cardiac output is significantly reduced. A shorter APD is likely to form a reentrant wave, but the correlation with the complexity of the reentrant wave has not been elucidated. However, as observed in Figure 3 and Table 1, the APD has significant influences on both the SV and tension, as indicated by statistically significant correlations.

In this study, we have implemented the ventricular tachyarrhythmia condition by changing the conductance of *I*_*Ks*_ from its normal value to 100-fold. Even though we increased *g*_*Ks*_ up to 100-fold to simulate extreme situations, the APD under the 100-fold-increased *g*_*Ks*_ condition was 90 ms in the sinus rhythm and 74 ms during reentry (Supplementary Figure S1). It is similar to the APDs when the KCNJ2 E299V (72–90 ms) and KCNQ1 V241F mutations (76 ms) are expressed (Cerrone et al., 2013; Heikhmakhtiar et al., 2018). In their experiment, Banville et al. successfully observed the APD variation during ventricular tachycardia. They reported an average APD of 107 ± 7 ms during ventricular tachycardia, which means our simulation results include the ventricular tachycardia condition (Banville et al., 2004).

The dominant frequency at the time of tachyarrhythmia is higher than that during the sinus rhythm owing to the automatic depolarization caused by reentrant waves. If the dominant frequency is above normal, the ventricle does not have enough time to fully expand and become filled with blood; thus, it is not possible for it to eject enough blood at the time of contraction. This mechanism applies not only to the sinus rhythm but also to the reentrant wave condition. In our previous study, we found a correlation between the dominant frequency and ventricular ejection capacity during tachyarrhythmia (Jeong and Lim, 2018a). Consistent with these results, we confirmed in this study that the higher the dominant frequency, the lower is the ventricular ejection capacity based on how the dominant frequency affects the ampTens.

As shown in prior studies, the ends of a filament, such as an O-type filament, may be present only in the internal tissue of the ventricle, rather than on the surface of the ventricle (epicardium to epicardium, epicardium to endocardium, or endocardium to endocardium) (Clayton et al., 2006). In such a case, because a filament is detected therein even if the PS is not present, the number of filaments can be increased without changing the number of PSs. Furthermore, because the shape of a filament is bent or fragmented at the boundary of the ventricular tissue during reentry break-up, the morphology of the filaments inside the tissue may be more complicated, even though PSs are present only at certain positions. Therefore, in this study, a regression model has been created by distinguishing the PSs and filaments as separate, independent predictors.

Accordingly, among the four independent variables, the influence of the APD on the myocardial ampTens and SV is the statistically most significant (standardized beta coefficient: 0.859 of SV, 0.930 of ampTens). The next most influential factors are the dominant frequency (standardized beta coefficient: −0.809 of SV, −0.907 of ampTens) and filament (standardized beta coefficient: 0.713 of SV, 0.507 of ampTens). The PS has a statistically significant effect on the SV (standardized beta coefficient = 0.305, *p*-value < 0.05), but it has no significant effect on the myocardial ampTens (*p*-value = 0.161).

We have predicted the SV and myocardial ampTens using the multiple regression models considering the APD, dominant frequency, PS, and filament. However, the collinearity indices of the APD and dominant frequency, i.e., VIF values, are 79.032 and 71.137, respectively, so there is multicollinearity between the two predictors. Multicollinearity means that a certain independent variable A has a higher correlation with another independent variable C than it does with a dependent variable B. In a collinearity test, the VIF index can be used to determine the presence of multicollinearity. Here, the permissible maximum VIF index is 10. If the VIF indices of any independent variables are 10 or higher, the prediction result of the multiple regression model can be distorted by the multicollinearity between the independent variables (Akinwande et al., 2015). Because the VIF of the APD and dominant frequency are much higher than the permissible value of 10, we have assumed that the APD has a strong correlation with the dominant frequency, which is also confirmed by the significant correlation between them shown in Figure 3 and Table 1.

In this regression model with multicollinearity, it is impossible to adequately and quantitatively predict the influence of each independent variable on a dependent variable. Therefore, we have eliminated the APD (with the highest VIF index) from the independent variables and rederived the multiple regression model. In the regression model excluding the APD, the VIF of the dominant frequency has significantly reduced to 2.992. This has reconfirmed that a strong correlation exists between the APD and dominant frequency.

Myocardial tissue composed of cells with a shortened APD has a shorter conduction wavelength than that of myocardial tissue composed of normal cells, and the rotational rate of reentry becomes faster in the former. This causes rapid oscillation of the action potentials of ventricular tissue cells, leading to an increase in the mean frequency of the entire ventricle. Thus, the dominant frequency is more directly related to the APD than to the contractility of the heart. This relationship between these two electrical parameters indicates multicollinearity. Eventually, this means that the stochastically predicted SV and myocardial ampTens predicted using the multiple regression models, namely Model 1 and Model 3, may be distorted by the multicollinearity between the APD and dominant frequency.

In addition, there is also a direct correlation between the PS detected at the center of the reentry rotor and filament present within ventricular tissue during tachyarrhythmia, which indicated slight multicollinearity between these two electrical parameters. However, their VIF values are below the permissible maximum value of 10. Therefore, we have considered that the multicollinearity between the PS and filament does not significantly affect the predictions of the SV and myocardial ampTens in any of the models (Models 1 through 4).

As a result, using the multiple regression models with the dominant frequency, PS, and filament as predictors based on the statistical results, we were able to quantitatively determine the extent to which each independent variable affected the SV and ampTens. Even though the same set of electrical parameters is considered, the influence of the electrical parameters changes depending on the mechanical parameters being evaluated. The filament has the highest effect on the SV (standardized beta coefficient = 0.853), and the dominant frequency has the highest effect on the myocardial ampTens (standardized beta coefficient = −0.813).

In this study, we focused on the statistical analysis of electrical and mechanical parameters and chose the stochastic model using three electrical parameters, which are dominant frequency, PS and filaments, as the optimal model for predicting mechanical performance during ventricular tachyarrhythmia. However, from a physiological point of view, the APD is the most influential electrical parameter (Hille, 1978). Furthermore, individual linear correlations between the APD and mechanical parameters are the highest. Accordingly, the accuracy of the multiple regression model including the APD (Models S1 and S2 in Supplementary Material) is higher than that of the multiple regression model including the dominant frequency (*R*^2^ scores: 0.866 for SV and 0.885 for ampTens; Supplementary Tables S1, S2). In these models, the APD has the greatest effect on the myocardial ampTens (standardized beta coefficient: 0.884), but the number of filaments has the greatest effect on the SV (standardized beta coefficient: 0.752).

Despite the direct association between the PS and filament, their correlations with the SV and ampTens are contradictory in the case of ventricular tachyarrhythmia. The PS is proportional to the number of rotors, that is, the number of reentrant waves because it corresponds to the center of the reentry rotor during tachyarrhythmia. However, as mentioned earlier, filaments can be present within the tissue even without reentrant waves on the ventricular surface; hence, filaments are related to the size of the vortex, that is, to the length of the vortex rather than to the number of rotors. The number of reentrant waves is related to the complexity of the waves because it is increased by the reentrant break-up, but there is no close correlation between the reentrant break-up and magnitude of the vortex. The long length of the vortex in the reentrant waves means that there are many myocardial tissues in the same state, indicating that the ventricular tissue cells are synchronously contracting. In contrast, ventricular tachyarrhythmia increases the severity of mechanical contractility when the asynchronous contraction of ventricular tissue cells occurs. Accordingly, the extended length of the vortex (the increased number of filaments) during ventricular tachyarrhythmia has a different meaning regarding the severity of mechanical contractility.

The myocardial tension refers to the myocyte-level contractility. However, the stroke volume is a global metric reflecting the organ-level contractility and is affected by the myocardial tension. In general, there is a proportional correlation as follows: tension/after loads ∼ stroke volume. We demonstrated this correlation from the correlation analysis and Pearson correlation coefficients, which was 0.887 (*p*-value < 0.05 in Table 1). Furthermore, the ampTens was obtained by integrating the tension of whole ventricular cells. Therefore, the ampTens can reflect the organ-level contractility.

Generally, it is assumed that linear relationships exist between the parameters to construct the linear regression models used to determine the individual and integrative correlations between the electrical and mechanical parameters during ventricular tachyarrhythmia. However, not all the electrical parameters used in regression analysis have linear relationships with the mechanical parameters. To improve the prediction accuracy of the regression model, we should consider the non-linear relationships between parameters in the future. Another assumption is that only cardiac electrical activation affects mechanical contraction. We did not consider mechanoelectrical feedback such as stretch-activated channels. For real-world scenarios, we need to use improved models including not only electromechanical properties but also mechanoelectrical feedback.

In the excitation–contraction coupling mechanism of cardiomyocytes, calcium is involved in the activation of electrical action potentials and the generation of tension. when the cell is depolarized, calcium introduced into the cell and induces the release of calcium from the JSR to increase the intracellular calcium concentration. Some of the intracellular calcium binds to Troponin C to form cross-bridge. Therefore, calcium bound to Troponin C is an important link between the electrical activity of cardiomyocytes and the generation of tension (Ji et al., 2015). Some models including the ventricular models suggested by Shannon et al. (2004) and Grandi et al. (2010) take this dynamic calcium buffer into account. However, in this study, the extracted calcium information from the electrophysiological simulation is the general calcium buffer. To improve the results, the model implementing the dynamic intracellular calcium buffers is needed.

## Conclusion

We have confirmed that not only the APD but also other electrical parameters such as the dominant frequency, PS, and filaments can affect mechanical contractility during ventricular tachyarrhythmia. In the absence of other electrical parameters, the APD has the greatest effect on mechanical contractility. Furthermore, even though the same set of electrical parameters are considered, the influence of the electrical parameters changes depending on the mechanical parameters being evaluated. From a statistical point of view, the filament has the greatest effect on the SV, and the dominant frequency has the greatest effect on the myocardial ampTens. Hence, it is necessary to consider these results for future studies on ventricular tachyarrhythmia.

## Data Availability Statement

All datasets generated for this study are included in the article/Supplementary Material.

## Author Contributions

This manuscript is the intellectual product of the entire team. All the authors contributed (to varying degrees) toward the analyses performed, developing the research concept, simulation design, developing the simulation source code, performing the simulation, and writing of the manuscript.

## Conflict of Interest

The authors declare that the research was conducted in the absence of any commercial or financial relationships that could be construed as a potential conflict of interest.
