# Supplementary material for: Relationship Between Electrical Instability and Pumping Performance During Ventricular Tachyarrhythmia: Computational Study
Source: Front Physiol. 2020 Mar 24;11:220. doi: 10.3389/fphys.2020.00220 (PMC7105731; doi:10.3389/fphys.2020.00220)
Supplement: Supplementary file 1 [file Data_Sheet_1.PDF]

Supplementary Material

Relationship between Electrical Instability and Pumping Performance during Ventricular Tachyarrhythmia: Computational Study

Da Un Jeong[1], Ki Moo Lim[2*]

*** Correspondence:** Ki Moo Lim: kmlim@kumoh.ac.kr

1 3D human ventricular modeling

To mimic the various ventricular tachyarrhythmia conditions, we used the validated 3-dimensional human ventricular model. The human ventricular model was implemented by solving the complex equations, which are based on continuum mechanics and reaction-diffusion systems to compute the cardiac tissue and whole-heart electromechanics. Numerical solution of these equations is derived from the framework of finite element methods (FEM), which requires a reconstruction of heart geometry and fiber structure of the myocardial preparation(Fijoy et al., 2010). We reconstructed the human ventricular model based on publicly available magnetic resonance imaging (MRI) with both fiber orientation information and cardiac tissue heterogeneity information. First, we performed the level-set segmentation on the MRI stack to separate myocardium from the suspension media. For level-set segmentation, we used the segmentation function of ITK program. The segmentation was achieved through the evolution of a surface Γ, which was implicitly represented as the zero level set of time-dependent 3D function Φ(x, y, z, t).

$$\Gamma(\mathrm{t}) = \{x, y, z | \Phi(x, y, z, t) = 0\} \qquad (1)$$

Where x, y, z are the Cartesian coordinates, and t is the time. For the implementation of the level set segmentation, the evolution of Φ was as follows:

$$\Phi = \begin{cases} \Phi_t = \alpha P(x, y, z)|\nabla\Phi| + \beta k(x, y, z)|\nabla\Phi| \\ \Phi(x, y, z), \qquad t = 0 \end{cases} \qquad (2)$$

Where Φ_t is the partial derivation of Φ with respect to time. α and β are constants whose values are experimentally determined. P(x, y, z) is the propagation term, which consists of the 3D function I(x, y, z) representing the intensity of the 3D image to be segmented. U is the upper intensity limit and L is the lower intensity limit. U and L are defined by selecting region in the input image. k(x, y, z) is the mean curvature term, defined as the divergence of gradient of Φ. This is for smoothing the evolving surface.

Second, the ventricles were detached from the atria. Third, in order to create the atrioventricular boundary, we manually marked the landmark points around ventricles along the atrioventricular border on each tenth slide in the MRI stack. Fourth, a 3D cubic Hermite was aligned along the landmark points of the border, generation a surface of the atrioventricular boundary. We used the surface mesh as a guideline for generating the finite element mesh of the ventricular model.

Fiber and laminar sheet structural information of the ventricles were obtained from the diffusion tensor MRI (DTMRI) data set. In order to incorporate fiber and laminar sheet structure in the ventricular model, tensor and tensor gradient were defined at each node of the finite element mesh and interpolated using Hermite interpolation (Gurev et al., 2011). The tensor eigenvectors from the interpolated tensor field represent the fiber and laminar sheet structured heart, as previously verified (Helm et al., 2005).

In order to implement the heterogeneous tissue, we assumed that conductance varied among the parts of the ventricles, which are endocardium, mid-myocardium, and epicardium, as shown in the left of Figure 2 A. To distinguish each part, we given electrical stimulation to the sub-endocardial surface and the endocardial surface, respectively. We determined the endocardium and epicardium as the tissues where the stimulus was propagated for 2 ms, and the mid-myocardium as the middle tissues, which was neither endocardium nor epicardium. Since the implemented ventricular tissue is anisotropic, we set the anisotropy ratio of conduction velocity, which are measured longitudinal to transverse, to 1.5.

2 Supplementary Figures and Tables

2.1 Supplementary Figures

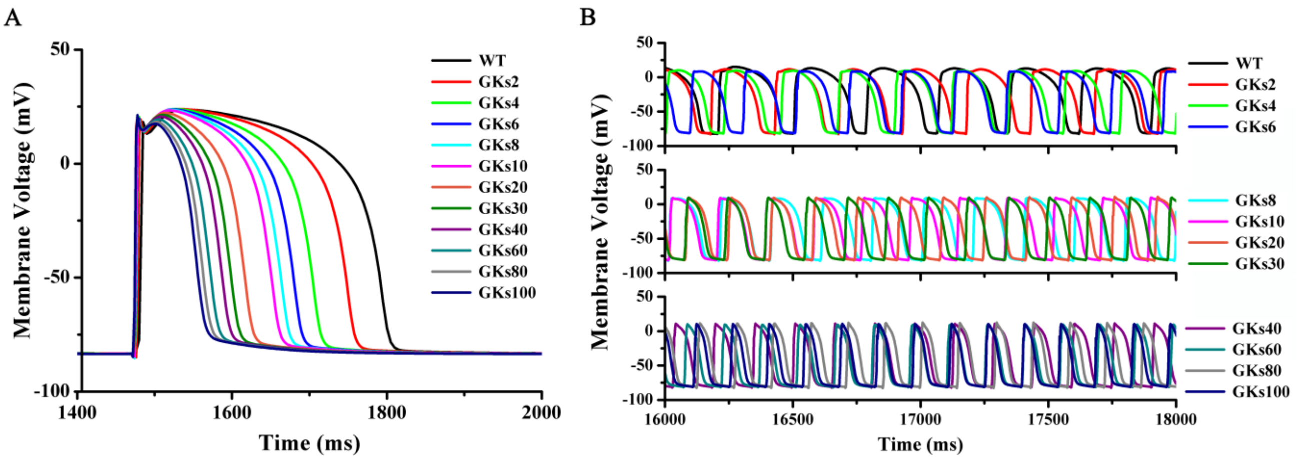

Supplementary Figure 1. Action potential shapes according to the electrical conductance variation of I_{Ks} channel; A, action potential shapes during 600 ms of one basic cycle length; B, action potential shapes during 2,000 ms of reentry; WT, wild type; GKs2, GKs4, GKs6, GKs8, GKs10, GKs20, GKs30, GKs40, GKs60, GKs80, and GKs100; the conditions that the electrical conductance increased doubled, 4-fold, 6-fold, 8-fold, 10-fold, 20-fold, 30-fold, 40-fold, 60-fold, 80-fold, and 100-fold, respectively

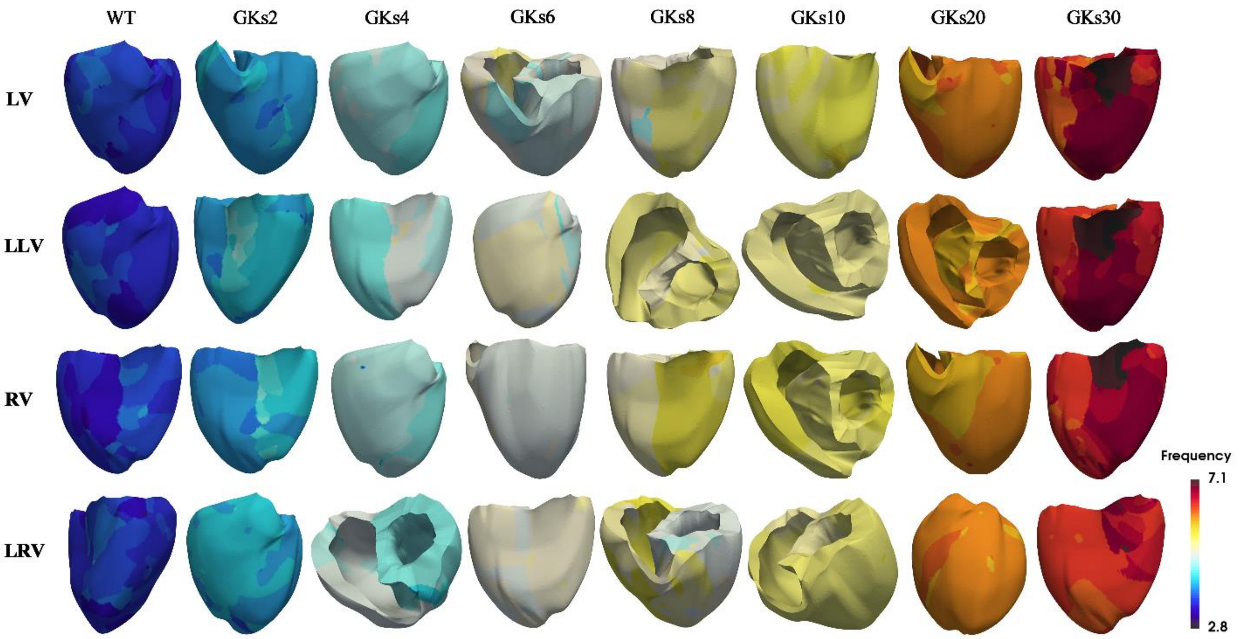

Supplementary Figure 2. Dominant frequency contours depending on the variation of electrical conductance of I_{Ks} channel. WT, wild type; GKs2, GKs4, GKs6, GKs8, GKs10, GKs20, GKs30; the conditions that the electrical conductance increased doubled, 4-fold, 6-fold, 8-fold, 10-fold, 20-fold, and 30-fold, respectively; We applied S2 stimulus on the whole left ventricle (LV), the lower part of left ventricle (LLV), the whole right ventricle (RV), and the lower part of right ventricle (LRV).

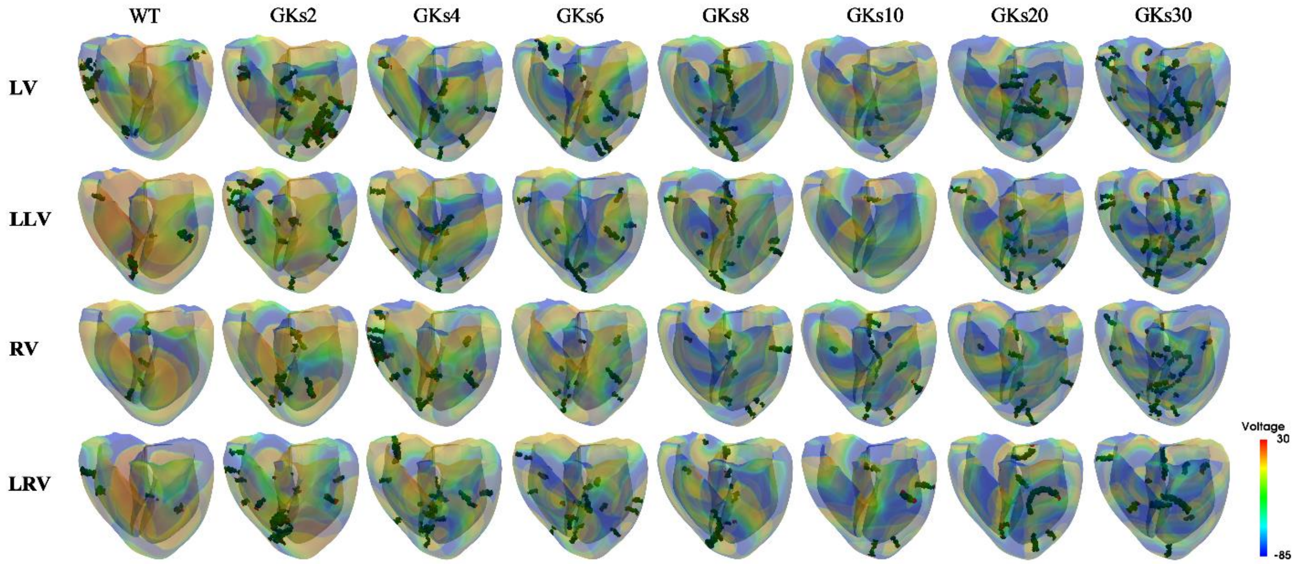

Supplementary Figure 3. Contours of membrane voltage with phase singularities and filaments. WT, wild type; GKs2, GKs4, GKs6, GKs8, GKs10, GKs20, GKs30; the conditions that the electrical conductance increased doubled, 4-fold, 6-fold, 8-fold, 10-fold, 20-fold, and 30-fold, respectively; We applied S2 stimulus on the whole left ventricle (LV), the lower part of left ventricle (LLV), the whole right ventricle (RV), and the lower part of right ventricle (LRV).

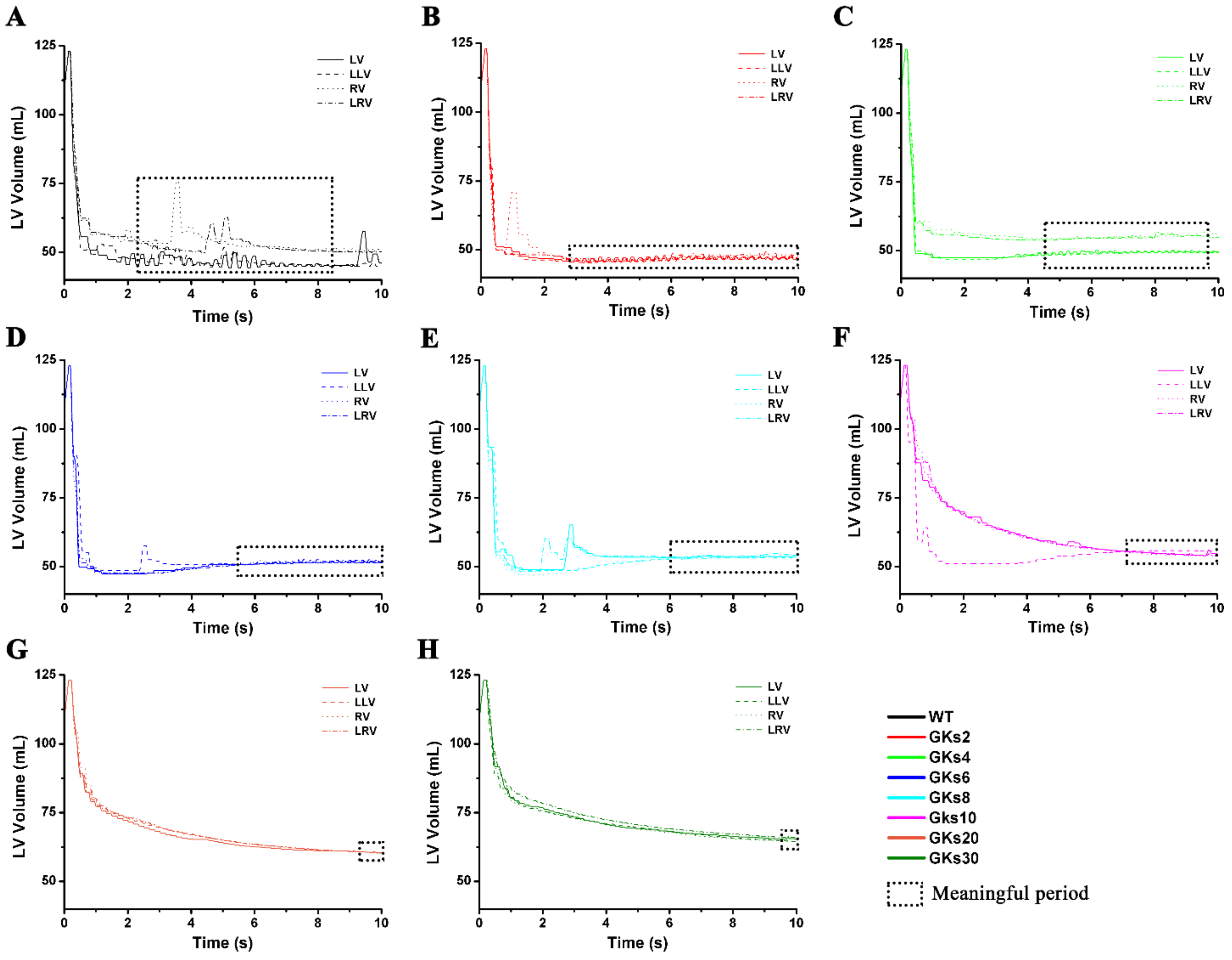

Supplementary Figure 4. Volume curves of left ventricles during ventricular tachyarrhythmia. WT, wild type; GKs2, GKs4, GKs6, GKs8, GKs10, GKs20, GKs30; the conditions that the electrical conductance increased doubled, 4-fold, 6-fold, 8-fold, 10-fold, 20-fold, and 30-fold, respectively; We applied S2 stimulus on the whole left ventricle (LV), the lower part of left ventricle (LLV), the whole right ventricle (RV), and the lower part of right ventricle (LRV).

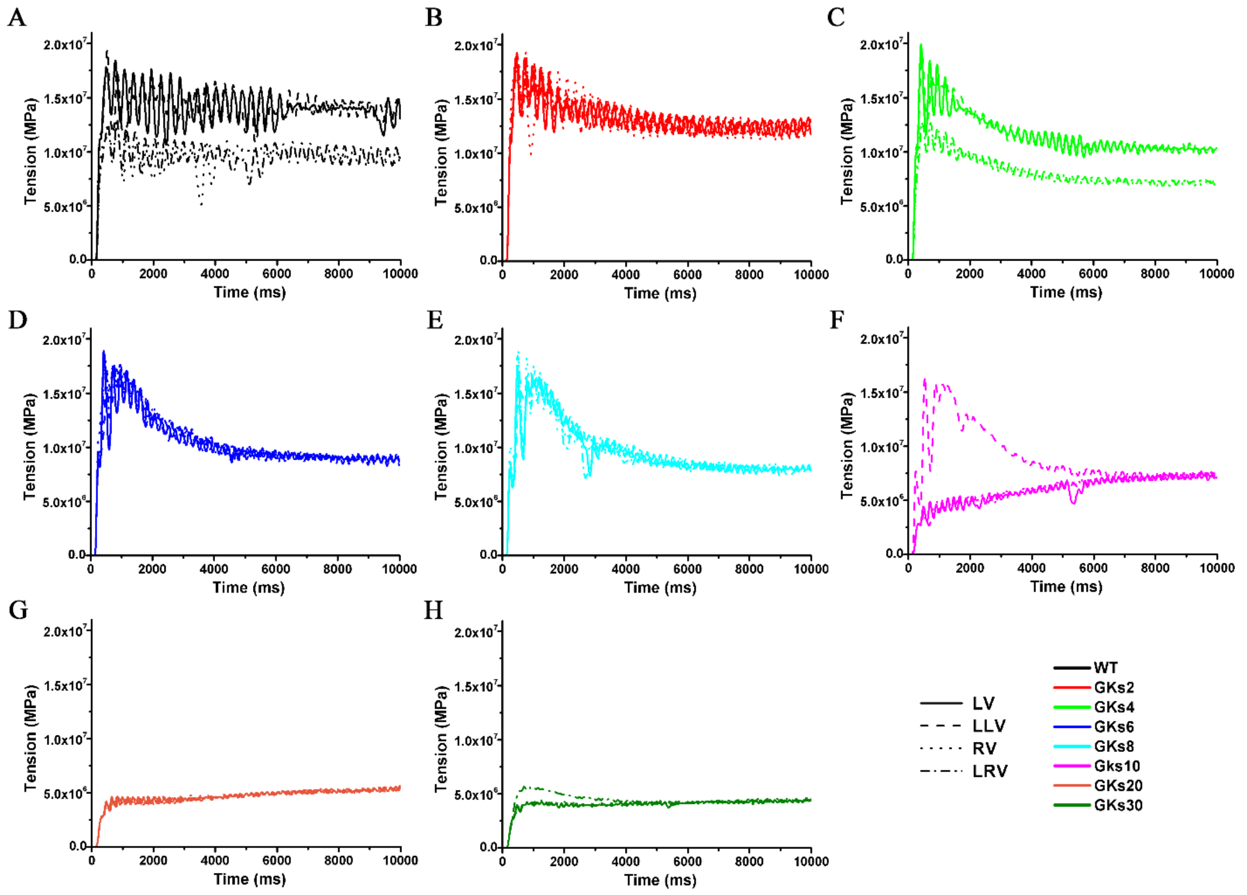

Supplementary Figure 5. Tension curves during ventricular tachyarrhythmia. WT, wild type; GKs2, GKs4, GKs6, GKs8, GKs10, GKs20, GKs30; the conditions that the electrical conductance increased doubled, 4-fold, 6-fold, 8-fold, 10-fold, 20-fold, and 30-fold, respectively; We applied S2 stimulus on the whole left ventricle (LV), the lower part of left ventricle (LLV), the whole right ventricle (RV), and the lower part of right ventricle (LRV).

2.2 Supplementary Tables

Supplementary Table 1. Regression model summary. Model S1 and S2 were to predict SV and ampTens, respectively, using three electrical variables (DF was excluded).

Model	R	Adjusted R^2	SE	R^2 change	F	df1	df2	Sig. F (p-value)	Durbin-Watson
S1	0.935	0.866	0.20517	0.875	102.269	3	44	0.000	1.502
S2	0.945	0.885	0.12912	0.892	121.531	3	44	0.000	1.936

Supplementary Table 2. Model S1 and S2 were to predict SV and ampTens, respectively, using three electrical variables (DF was excluded).

Model		Unstandardized coefficient		Standardized coefficient	t	Sig. t (p-value)	Collinearity Statistics	
		B	SE	beta			Tolerance	VIF
S1	(intercept)	-0.570	0.106		-5.392	0.000		
	APD	0.006	0.001	0.553	7.926	0.000	0.585	1.710
	PS	-0.010	0.002	-0.450	-4.791	0.000	0.323	3.099
	Filament	5.333E-5	0.000	0.752	6.815	0.000	0.234	4.270
S2	(intercept)	-0.378	0.066		-5.683	0.000		
	APD	0.007	0.000	0.884	13.656	0.000	0.585	1.710
	PS	-0.004	0.001	-0.279	-3.206	0.003	0.323	3.099
	Filament	1.055E-5	0.000	0.219	2.143	0.038	0.234	4.270

Supplementary Table 3. ANOVA test of multiple stochastic models. In Model 1, all of electrical variables were used to predict stroke volume. In Model 2, three electrical variables (dominant frequency, phase singularity, and filament) except for APD were used to predict stroke volume. In Model S1, three electrical variables (dominant frequency, phase singularity, and filament) except for DF were used to predict stroke volume. In Model 3, all of electrical variables were used to predict ampTens. In Model 4, three electrical variables except APD were used to predict ampTens. In Model S2, electrical variables except for DF were used to predict ampTens.

Model		Sum of Squares	df	Mean Square	F	Sig. F (p-value)
1	Regression	13.305	4	3.326	97.845	0.000
	Residual	1.462	43	0.34		
	Total	14.767	47			
2	Regression	12.570	3	4.190	83.932	0.000
	Residual	2.197	44	0.05		
	Total	14.767	47			
S1	Regression	12.915	3	4.305	102.269	0.000
	Residual	1.852	44	0.042		
	Total	14.767	37			
3	Regression	6.134	4	1.534	97.290	0.000
	Residual	0.678	43	0.016		

	Total	6.812	47			
4	Regression	5.891	3	1.964	93.834	0.000
	Residual	0.921	44	0.021		
	Total	6.812	47			
S2	Regression	6.079	3	2.026	121.531	0.000
	Residual	0.734	44	0.017		
	Total	6.812	47			

* APD, action potential duration; ampTens, amplitude of myocardial tension; df, degree of freedom; F, F-statistics; Sig. F, significant value of F-statistics.

Supplementary Table 4. Parameters for electrophysiology model

Parameter	Definition	Value	Unit
R	Gas constant	8.3143	$J \cdot K^{-1} \cdot mol^{-1}$
T	Temperature	310	K
F	Faraday constant	96.4867	C/mmol
C_m	Cell capacitance per unit surface area	2	$\mu F/cm^2$
S	Surface-to-volume ratio	0.2	μm^{-1}
ρ	Cellular resistivity	162	Ω ·cm
V_C	Cytoplasmic volume	16,404	μm^3
V_{SR}	Sarcoplasmic reticulum volume	1,094	μm^3
K_o	Extracellular K^+ concentration	5.4	mM
Na_o	Extracellular Na^+ concentration	140	mM
Ca_o	Extracellular Ca^{2+} concentration	2	mM
G_{Na}	Maximal I_{Na} conductance	14.838	nS/pF
G_{K1}	Maximal I_{K1} conductance	5.405	nS/pF
$G_{to,}$ epi, M	Maximal epicardial I_{to} conductance	0.294	nS/pF
$G_{to,}$ endo	Maximal endocardial I_{to} conductance	0.073	nS/pF
G_{kr}	Maximal I_{Kr} conductance	0.096	nS/pF
$G_{Ks,}$ epi,endo	Maximal epi- and endocardial I_{Ks} conductance	0.245	nS/pF
$G_{Ks,}$ M	Maximal M cell I_{Ks} conductance	0.062	nS/pF
p_{KNa}	Relative I_{Ks} permeability to Na^+	0.03	
G_{CaL}	Maximal I_{Cal} conductance	1.75^{-4}	$cm^3 \cdot \mu F^{-1} \cdot s^{-1}$
k_{NaCa}	Maximal I_{NaCa}	1,000	pA/pF
γ	Voltage dependence parameter of I_{NaCa}	0.35	
K_{mCa}	Ca_i half-saturation constant for I_{NaCa}	1.38	mM
K_{mNai}	Na_i half-saturation constant for I_{NaCa}	87.5	mM
K_{sat}	Saturation factor for I_{NaCa}	0.1	
α	Factor enhancing outward nature of I_{NaCa}	2.5	
P_{NaK}	Maximal I_{NaK}	1.362	pA/pF
K_{mK}	K_O half-saturation constant of I_{NaK}	1	mM
K_{mNa}	Na_i half-saturation constant of I_{NaK}	40	mM
G_{pk}	Maximal I_{pK} conductance	0.0146	nS/pF
G_{pCa}	Maximal I_{pCa} conductance	0.025	nS/pF
K_{pCa}	Ca_i half-saturation constant of I_{pCa}	0.0005	mM
G_{bNa}	Maximal I_{bNa} conductance	0.00029	nS/pF
G_{bCa}	Maximal I_{bCa} conductance	0.000592	nS/pF
V_{maxup}	Maximal I_{up}	0.000425	mM/ms
K_{up}	Half-saturation constant of I_{up}	0.00025	mM
A_{rel}	Maximal Ca_{SR}-dependent I_{rel}	16.464	mM/s
B_{rel}	Ca_{SR} half-saturation constant of I_{rel}	0.25	mM
C_{rel}	Maximal Ca_{SR}-independent I_{rel}	8.232	mM/s
V_{leak}	Maximal I_{leak}	0.00008	ms^{-1}
Buf_c	Total cytoplasmic buffer concentration	0.15	mM
K_{bufc}	Ca_i half-saturation constant for cytoplasmic buffer	0.001	mM
Buf_{sr}	Total sarcoplasmic buffer concentration	20	mM
K_{bufsr}	Ca_{SR} half-saturation constant for sarcoplasmic buffer	0.3	mM

Supplementary Table 5. Parameters for mechanical model

Parameter	Definition	Value	Unit
SL_{max}	Maximal length specific examples the filament	2.4	μm
SL_{min}	Minimal length specific examples the filament	1.4	μm
$length_{thick}$	Thick filaments length	1.65	μm
$length_{hbare}$	Bare zon length	0.1	μm
$length_{thin}$	Thin filaments length	1.2	μm
TmpC	Environmental temperature rage	Range=15-37	℃
Qk_{on}	k_{on} over temperature change	1.5	
Qk_{off}	k_{off} over temperature change	1.3	
Qk_{n_p}	k_{n_p} over temperature change	1.6	
Qk_{p_n}	k_{p_n} over temperature change	1.6	
Qf_{app}	f_{app} over temperature change	6.25	
Qg_{app}	g_{app} over temperature change	2.5	
Qh_{f}	h_{f} over temperature change	6.25	
Qh_{b}	h_{b} over temperature change	6.25	
Qg_{xb}	g_{xb} over temperature change	6.25	
k_{on}	The complete base rate constant for binding under default conditions	50	$\mu M^{-1} s^{-1}$
k_{offL}	The complete base rate constant for unbinding the low affinity sites.	250	s^{-1}
k_{offH}	The complete base rate constant for unbinding from high-affinity sites	25	s^{-1}
$perm_{50}$	The half-activation constant	0.5	
n_{perm}	The Hill coefficient	150	
K_{p_n}	The permissive to nonpermissive transition rate In RU state	500	s^{-1}
f_{app}	The crossbridge forward transition rates	500	s^{-1}
g_{app}	The crossbridge reverse rates	70	s^{-1}
gslmod	Scale the effects of the thick-filament	6	
h_{f}	The forward transition rate h_f between the strongly-bound states XB_{PreR} to XB_{PostR}	2000	s^{-1}
hfmdc	The extent to which mean strain of the prerotated state affects the isomerization rate	5	
h_{b}	The backward transition rate from XB_{PostR} to XB_{PreR}	400	s^{-1}
g_{xb}	The ATP-consuming detachment transition rate	70	s^{-1}
σ_p	The effects of strain for positive shortening velocities	8	
σ_n	The effects of strain for negative shortening velocities	1	
x_0	Mean strain of strongly-bound states	0.007	μm
φ	An empirically derived scaling term	2	
SL_{rest}	Sarcomere length in rest state	1.9	μm
$PCon_{titin}$	The contribution rate to passive strength of titin	0.002	(Unit normalized force)
$PExp_{titin}$	The exponential to passive strength of titin	10	
$SL_{collagen}$	The maximal Sarcomere length by collagen	2.25	μm
$PCon_{collagen}$	The contribution rate to passive strength of titin	0.02	(Unit normalized force)
$PExp_{collagen}$	The exponential to passive strength of collagen	70	
Mass	Mass element each animal	0.00005(rat) 0.00025(rabbit)	$s^2 \mu m^{-1}$
Viscosity	The mean value found experimentally from Newtonian viscosity element	0.003	$s \mu m^{-1}$
$F_{afterload}^{constant}$	if isotonic contraction (after release)	Range= 0.0-1.0	(Unit normalized force)
KSE	The stiffness in units of normalized force per μm	Range=1.0-200.0	μm^{-1}

Fijoy, V., Hermenegild, A., Anton J., P., Junjie, C., Ferdinand, K., Peter, K., et al. (2010). Image-based models of cardiac structure in health and disease. *Wiley Interdiscip. Rev. Syst. Biol. Med.* 2, 489–506. doi:10.1002/wsbm.76.

Gurev, V., Lee, T., Constantino, J., Arevalo, H., and Natalia, A. (2011). Models of Cardiac electromechanics based on indivisual hearts imaging data: image-based electromechanical models of the heart. *Biomech. Model. Mechanobiol.* 10, 295–306. doi:10.1007/s10237-010-0235-5.Models.

Helm, P., Beg, M. F., Miller, M., and Raimond, L. (2005). Measuring and Mapping Cardiac Fiber and Laminar Architecture Using Diffusion Tensor MR Imaging Topics Review of DTMRI and estimation of cardiac fiber. *Ann. N. Y. Acad. Sci.* 1047, 296–307.
